# One-Year Mortality after Hemodialysis Initiation: The Prognostic Role of the CHA_2_DS_2_-VASc Score

**DOI:** 10.3390/jcm12031011

**Published:** 2023-01-28

**Authors:** Ana Mafalda Abrantes, Bernardo Marques da Silva, Carolina Branco, Cláudia Costa, Nadiesda Peres, Ana Cardoso, Mariana Sant’Ana, José Agapito Fonseca, Cristina Outerelo, Cristina Resina, José António Lopes, Joana Gameiro

**Affiliations:** 1 Division of Internal Medicine II, Centro Hospitalar Universitário Lisboa Norte, EPE, Av. Prof. Egas Moniz, 1649-035 Lisboa, Portugal; 2 Division of Nephrology and Renal Transplantation, Centro Hospitalar Universitário Lisboa Norte, EPE, Av. Prof. Egas Moniz, 1649-035 Lisboa, Portugal; 3 Faculdade de Medicina, Universidade de Lisboa, Av. Prof. Egas Moniz, 1649-035 Lisboa, Portugal

**Keywords:** hemodialysis, CHA2D2SVASc, mortality

## Abstract

Background: CKD is a significant cause of morbidity, cardiovascular and all-cause mortality. CHA2DS2-VASc is a score used in patients with atrial fibrillation to predict thromboembolic risk; it also appears to be useful to predict mortality risk. The aim of the study was to evaluate CHA2DS2-VASc scores as a tool for predicting one-year mortality after hemodialysis is started and for identifying factors associated with higher mortality. Methods: Retrospective analysis of patients who started hemodialysis between January 2014 and December 2019 in Centro Hospitalar Universitário Lisboa Norte. We evaluated mortality within one year of hemodialysis initiation. The CHA2DS2-VASc score was calculated at the start of hemodialysis. Results: Of 856 patients analyzed, their mean age was 68.3 ± 15.5 years and the majority were male (61.1%) and Caucasian (84.5%). Mortality within one-year after starting hemodialysis was 17.8% (n = 152). The CHA2DS2-VASc score was significantly higher (4.4 ± 1.7 vs. 3.5 ± 1.8, *p* < 0.001) in patients who died and satisfactorily predicted the one-year risk of mortality (AUC 0.646, 95% CI 0.6–0.7, *p* < 0.001), with a sensitivity of 71.7%, a specificity of 49.1%, a positive predictive value of 23.9% and a negative predictive value of 89.2%. In the multivariate analysis, CHA2DS2-VASc ≥3.5 (adjusted HR 2.24 95% CI (1.48–3.37), *p* < 0.001) and central venous catheter at dialysis initiation (adjusted HR 3.06 95% CI (1.93–4.85)) were significant predictors of one-year mortality. Conclusion: A CHA2DS2-VASc score ≥3.5 and central venous catheter at hemodialysis initiation were predictors of one-year mortality, allowing for risk stratification in hemodialysis patients.

## 1. Introduction

Chronic kidney disease (CKD) is defined as abnormalities in the kidneys’ structure or function for more than three months [1]. The prevalence of CKD has increased in recent decades [1,2]. This condition is recognized as a relevant cause of morbidity, cardiovascular and all-cause mortality. These patients have multiple cardiovascular risk factors including hypertension, diabetes, dyslipidemia and smoking [3]. The risk of death increases with kidney disfunction and results mainly from cardiovascular death [4]. Throughout the course of the disease, cardiac and vascular remodeling, arterial stiffness, accelerated atherosclerosis, ischemic cardiomyopathy, heart failure and kidney disease progression contribute to cardiovascular mortality [5]. The identification of CKD patients with a higher mortality risk is crucial for developing health programs, therapeutic strategies and follow-up programs aimed at minimizing this risk [6].

CHA2DS2-VASc is a well-known score system usually applied in patients with atrial fibrillation to establish stroke and systemic embolization risk [7,8,9]. Given the great facility of its implementation, its efficiency and its low cost, it is expected to be a useful tool as a cardiovascular prognostic marker in several conditions. Previous studies have evaluated the application of this score in patients with heart failure with reduced ejection fraction [10], sick sinus syndrome [11] and stroke with prior coronary artery disease [12], regardless of the co-existence of atrial fibrillation. Additionally, it is also useful in patients without atrial fibrillation for predicting cardiovascular outcomes, including mortality [10,11,12]. Although the score was not initially designed to predict mortality, certain studies have demonstrated the ability of this score to predict mortality in patients with acute coronary syndrome [13,14], as well as in patients undergoing angioplasty [15]. Few studies have evaluated the effectiveness of this score as a mortality risk predictor in patients with CKD—particularly in patients starting renal replacement therapy [6,16,17].

We aimed to evaluate the role of the CHA2DS2-VASc score in predicting one-year mortality after starting hemodialysis and in identifying factors associated with higher mortality.

## 2. Methods

This study is a retrospective analysis of patients who initiated hemodialysis between January 2014 and December 2019 in Centro Hospitalar Universitário Lisboa Norte (CHULN) in Lisbon, Portugal. The Ethical Committee approved this study, in agreement with institutional guidelines. Informed consent was waived, given the retrospective and non-interventional nature of the study.

### 2.1. Participants

We selected as eligible any adult patient (≥18 years of age) with CKD who initiated hemodialysis from 1 January 2014 to 31 December 2019. Patients with previous renal replacement therapy (RRT)—namely, peritoneal dialysis or renal transplant—were excluded, as were patients lost to follow-up.

### 2.2. Variables and Outcomes

Data was obtained from individual electronic clinical records. The following variables were collected: demographic characteristics (age, gender, race); comorbidities (CKD, diabetes mellitus, hypertension, ischemic cardiomyopathy, heart failure, chronic obstructive pulmonary disease (COPD), cerebrovascular disease, dementia, chronic liver disease, rheumatologic disease and previous or active malignancy); hemodialysis access (type of access); laboratory results at hemodialysis initiation (hemoglobin, serum albumin, serum ferritin, serum parathyroid hormone (PTH), SCr, estimated glomerular filtration rate (eGFR) and serum urea). The CHA2DS2-VASc score was calculated at hemodialysis initiation.

We evaluated all-cause mortality within one year of hemodialysis intiation.

### 2.3. Definitions

The estimated glomerular filtration rate (eGFR) was calculated using the Chronic Kidney Disease Epidemiology Collaboration (CKD-EPI) creatinine equation [18].

The presence of CKD was defined as an eGFR lower than 60 mL/min/1.73 m^2^.

Diabetes mellitus was defined in accordance with the American Diabetes Association Guidelines [19]. Arterial hypertension was diagnosed according to the European Society of Cardiology and European Society of Hypertension Guidelines [20]. For the remaining comorbidities, indication on clinical records of a previous diagnosis was considered sufficient.

The CHA2DS2-VASc score was calculated based on a scoring system, as follows: 1 point was assigned for congestive heart failure, hypertension, age between 65 and 74 years, diabetes mellitus, female sex and vascular disease, and 2 points were assigned for a history of stroke and age ≥ 75 years [7].

### 2.4. Statistical Methods

Categorical variables were described as the total number and percentage of each category, while continuous variables were described as the mean ± standard deviation. The Kolmogorov–Smirnov normality test was used to examine if variables were normally distributed. Continuous variables were compared using the Student’s *t*-test, whereas categorical variables were compared using the Chi-square test.

The Cox regression method was used to determine which variables could be predictive of mortality within the first year of hemodialysis initiation. Variables that were statistically significant in the univariate analysis were included in the multivariate analysis. Variables included in the CHA2DS2-VASc score were not included in the multivariate analysis to avoid collinearity.

The discriminatory ability for CHA2DS2-VASc to predict mortality within one year from dialysis initiation was determined using the receiver operating characteristic (ROC) curve. The cut-off value was defined as that with the highest validity by determining the Youden point. Calibration was tested using the Hosmer–Lemeshow test.

A Kaplan–Meier survival analysis was performed to estimate survival during the first year of hemodialysis, according to the CHA2DS2-VASc score.

Data were conveyed as hazard ratios (HR) with 95% confidence intervals (CI). Statistical significance was established as a *p*-value lower than 0.05. Statistical analyses were carried out using the statistical software package SPSS for Windows (version 21.0).

## 3. Results

In this cohort, 856 patients started hemodialysis as their first RRT. The baseline characteristics and outcomes are shown in Table 1.

The mean age was 68.3 ± 15.5 years and the majority of patients were male (61.1%) and Caucasian (84.5%). Concerning comorbidities, the most prevalent were hypertension (89.7%), diabetes (45.7%), ischemic cardiomyopathy (23%), neoplasia (20.3%), peripheral artery disease (16.7%), atrial fibrillation (16.0%) and cerebrovascular disease (16.0%). Other comorbidities had a lower prevalence—namely, COPD (10.4%), rheumatologic disease (8.4%), dementia (5%) and chronic liver disease (4.1%).

At dialysis initiation, the mean eGFR was 9.3 ± 5.8 mL/min/1.73m^2^, mean serum urea was 196.7 ± 80.8 mg/dL, mean hemoglobin was 9.7 ± 1.6 g/dL, the mean neutrophil/lymphocyte (N/L) ratio was 5.6 ± 1.8, mean albumin was 3.4 ± 0.7 g/dL, mean PTH was 352.9 ± 289.1 ng/mL and mean ferritin was 527.8 ± 196.9 ng/mL. Regarding vascular access at dialysis initiation, 60.9% of patients (n = 522) used a central venous catheter, 36.2% of patients (n = 310) had an arteriovenous fistula and 2.8% (n = 24) had an arteriovenous graft. The calculated mean CHA2DS2-VASc score was 3.7 ± 1.8.

### 3.1. One-Year Mortality

At a one-year follow-up after hemodialysis initiation, mortality was 17.8% (n = 152).

Patients who died within one year of starting hemodialysis were significantly older (*p* < 0.001), were more frequently Caucasian (*p* = 0.011) and were more likely to have pre-existing ischemic cardiomyopathy (*p* < 0.001), peripheral artery disease (*p* = 0.039), dementia (*p* = 0.028) and neoplasia (*p* = 0.043). At dialysis initiation, these patients had higher eGFR (*p* = 0.004), lower serum urea (*p* = 0.025), a higher N/L ratio (*p* = 0.019) and lower albumin (*p* < 0.001). Patients who started dialysis with a central venous catheter (*p* < 0.001) had a higher risk of one-year mortality. The CHA2DS2-VASc score was significantly higher (4.4 ± 1.7 vs. 3.7 ± 1.8, *p* < 0.001) in patients who died.

### 3.2. CHA2DS2-VASc Score and One-Year Mortality

**Figure 1:**
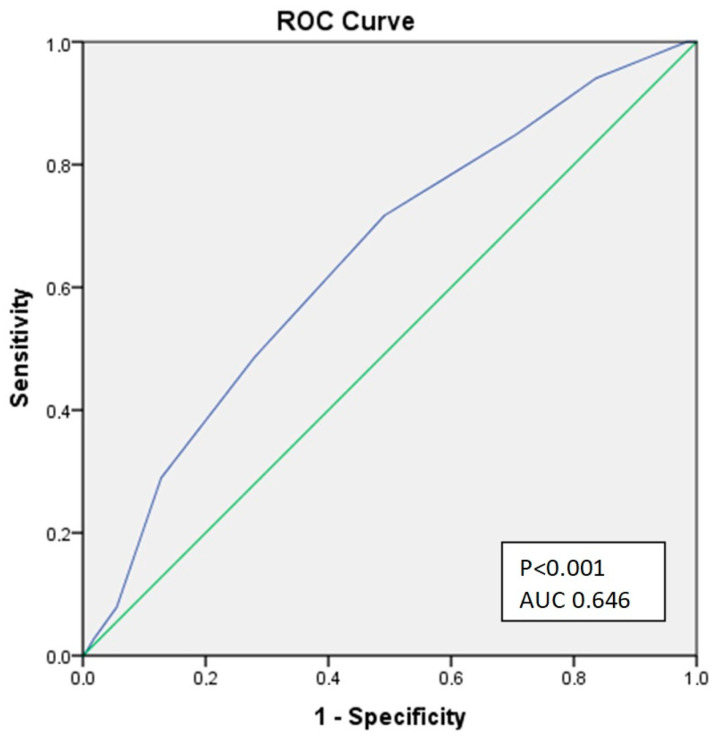
AUROC CHA2DS2-VASc one-year mortality.

The CHA2DS2-VASc score satisfactorily predicted the one-year risk of mortality, with an unadjusted hazard ratio of 1.32 (95% CI (1.20–1.46), *p* < 0.001). A ROC curve was produced to assess the discriminative ability of the CHA2DS2-VASc score for one-year risk mortality. The AUC for mortality prediction was 0.646 (*p* < 0.001 95% CI (0.6–0.7)), with a sensitivity 71.7% and a specificity of 49.1% (Figure 1).

The optimal CHA2DS2-VASc score cut-off was 3.5, which had a positive predictive value of 23.9% and a negative predictive value of 89.2%—which means that almost 90% of patients with a CHA2DS-VASc < 3.5 survived the first year after hemodialysis initiation.

Patients with a CHA2DS2-VASc score ≥ 3.5 were older (59.9 ± 16.1 vs. 75.7 ± 10.4 years, *p* < 0.001), less frequently male (51.9% vs. 71.8%, *p* < 0.001), more frequently Caucasian (91.9% vs. 76.5%, *p* < 0.001) and had a significantly higher prevalence of co-morbidities such as hypertension (95.8% vs. 83.3, *p* < 0.001), diabetes (65.4% vs. 23.5%, *p* < 0.001), ischemic cardiomyopathy (33.8% vs. 10.8%, *p* < 0.001), atrial fibrillation (28.6% vs. 1.5%, *p* < 0.001), COPD (14.0% vs. 6.3%, *p* < 0.001), cerebrovascular disease (28.6% vs. 1.5%, *p* < 0.001), peripheral artery disease (27.6% vs. 4.3%, *p* < 0.001) and dementia (8.8% vs. 0.7%, *p* < 0.001). Regarding laboratory tests at dialysis initiation, patients with a CHA2DS2-VASc score ≥ 3.5 had higher eGFR (9.9 ± 5.4 vs. 8.7 ± 6.2 mL/min/1.73 m^2^, *p* = 0.006), a higher N/L ratio (6.2 ± 2.5 vs. 4.9 ± 2.7, *p* = 0.001) and lower albumin (3.3 ± 0.7 vs. 3.5 ± 0.7 g/dL, *p* < 0.001). These patients were more likely to have started dialysis with a central venous catheter (67.8% vs. 53.2%, *p* < 0.001). Patient variables and outcomes are depicted in Table 2.

In the multivariate analysis, CHA2DS2-VASc ≥ 3.5 (adjusted OR 2.24 95% CI (1.48–3.37), *p* < 0.001) and having a central venous catheter at dialysis initiation (adjusted HR 3.06 95% CI (1.93–4.85)) were significant predictors of one-year mortality after hemodialysis initiation (Table 3).

**Figure 2:**
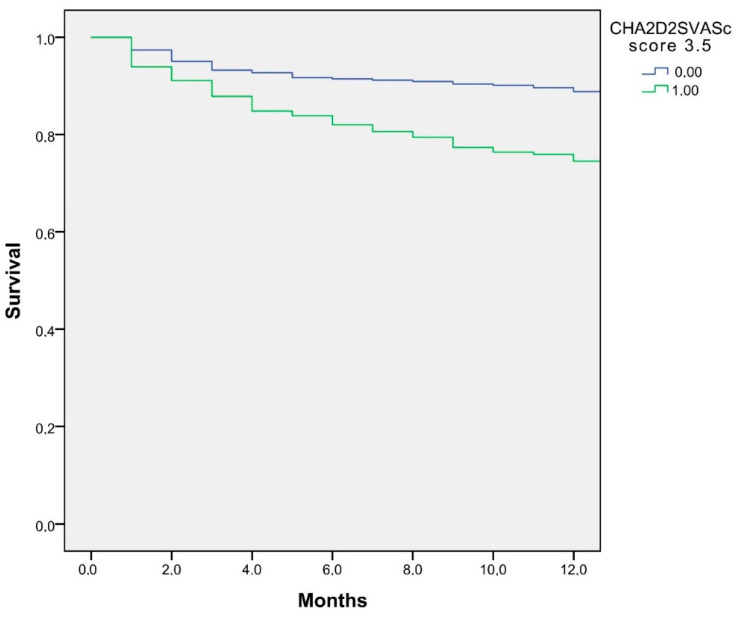
Kaplan–Meier curves displaying the estimated survival probability according to the CHA2DS2-VASc score (*p* < 0.001).

A Kaplan—Meier survival analysis is displayed in Figure 2. The one-year mortality survival was lower in patients with CHA2DS2-VASc ≥ 3.5 (76.1% vs. 89.3%, log-rank <0.001).

## 4. Discussion

In this retrospective study, the CHA2DS2-VASc score satisfactorily predicted the one-year risk of mortality. In fact, after adjustments for demographic and clinical variables, having a CHA2DS2-VASc ≥ 3.5 and a central venous catheter at dialysis initiation were significant predictors of one-year mortality.

The association of this score with mortality emphasizes the role of cardiovascular disease as a major cause of morbidity and mortality in patients on hemodialysis. Cardiovascular disease is present in 50–75% of hemodialysis patients, increasing their risk of cardiovascular mortality by 20 times [21,22]. Hypertension is present in more than 90% of patients and is one of the main risk factors for the development of coronary heart disease and ventricular hypertrophy [21]. Additional factors for cardiovascular disease include anemia, hyperhomocysteinemia, chronic kidney disease–mineral bone disorder (CKD–MBD), vascular calcification, oxidative stress, malnutrition and chronic inflammation [6,23]. There are also hemodialysis-related factors that contribute to cardiovascular disease—namely, volume overload, intradialytic hypotension and electrolyte imbalances—which may induce myocardial stunning and arrythmias [24].

According to the USRDS, mortality rates are 134 and 166 per 1000 patient-years for ESRD and hemodialysis patients, respectively. Cardiovascular disease is the main cause of mortality in ERSD patients [25,26]; arrhythmias, cardiac arrest, heart failure, stroke and atherosclerotic disease account for 48% of deaths [27]. Analyzing mortality in the first year of hemodialysis is of utmost importance as it is generally higher in this period, falling during the second year. Indeed, cardiovascular mortality during the first 4 months following the start of dialysis is 20 times higher than that of age-matched individuals in the US population [26]. The months after dialysis initiation are a critical period in which clinical vigilance of cardiovascular complications is vital. Thus, it is imperative to identify the most vulnerable patients.

The CHA2DS2-VASc score was first described in 2010 as a predictive scheme for stroke-risk stratification in patients with atrial fibrillation [7]. A recent study highlighted the role of CHA2DS2-VASc score as a predictor of mortality in patients with chronic kidney disease [6]. This study enrolled 437 patients with chronic kidney disease in stages three to five with a mortality of 37% and found that the CHA2DS2-VASc score significantly predicted cardiovascular and all-cause mortality. In another cohort of 87 non-dialysis CKD patients, CHA2DS2-VASc showed that it could be useful for assessing the risk of cardiovascular and all-cause mortality [16].

The CHA2DS2-VASc score has also been studied in other conditions, regardless of the existence of atrial fibrillation. In a prospective multicenter observational study including 1311 patients with heart failure with reduced ejection fraction, CHA2DS2-VASc and R2CHA2DS2-VASc significantly predicted mortality in this population [10]. Svendsen et al. applied this score to 1415 patients with sick sinus syndrome before pacemaker placement and found an association between the score and the occurrence of stroke and mortality [11].

In our cohort of hemodialysis patients, 17.8% died within the first year of hemodialysis initiation. These patients were older and had more cardiovascular comorbidities and other relevant conditions—namely, dementia and neoplasia. Additionally, the CHA2DS2-VASc score was significantly higher in patients who died. This score had a high sensitivity, which allows for the acknowledgement of patients with higher mortality risk. Despite the average specificity, the high negative predictive value means that almost 90% of patients with a CHA2DS2-VASc score < 3.5 have a low risk of mortality. Pradva et al. used the CHA2DS2-VASc score to predict a composite of cardiovascular outcomes (mortality, stroke or myocardial infarction) in a chronic hemodialysis cohort [25]. Our work has focused on one-year mortality and shows that the CHA2DS2-VASc score has discriminatory power for assessing this hard outcome.

We also identified that patients with CHA2DS2-VASc ≥ 3.5 had lower SCr, higher GFR, a higher N/L ratio and lower albumin values. Previous studies have already confirmed the association between lower SCr, lower albumin values and a higher N/L ratio at dialysis initiation and higher mortality [28,29,30,31,32]; this reflects the association between malnutrition, inflammation and cardiovascular disease and mortality.

Malnutrition in CKD patients is multifactorial and might result from a combination of inadequate food intake, dietary restrictions, increased inflammation, oxidant stress, increased nutrient losses in urine or dialysate, decreased levels of anabolic hormones or increased levels of catabolic hormones, metabolic acidosis, aging and physical deconditioning [33,34,35,36]. The malnutrition–inflammation–atherosclerosis (MIA) syndrome describes the association between malnutrition, inflammation and atherosclerosis that coexist in ESRD patients and contribute to cardiovascular mortality [33,37].

In this cohort, having a central venous catheter at hemodialysis initiation was an independent risk factor of one-year mortality in the multivariate analysis. This result reinforces previous studies associating this vascular access with increased mortality, independent of malnutrition and comorbidities [38,39,40].

This study presents some limitations: Firstly, this is a single center and retrospective study with a relatively small cohort of patients, which limits the generalization of the results. Secondly, the relatively low specificity of the risk score means that there is a significant number of false positives. Still, this risk score enables the recognition of high-risk patients. Thirdly, causes of mortality were not assessed.

Despite the mentioned limitations, our study presents significant relevance. To the best of our knowledge, this is the first study reporting the value of the CHA2DS2-VASc score for satisfactorily predicting one-year mortality in hemodialysis patients with a high sensitivity, considerable specificity and significant negative predictive value. A CHA2DS2-VASc score ≥ 3.5 is useful for stratifying hemodialysis patients according to their risk of mortality, as well as for defining preventive strategies for them. Additionally, we presented an extensive characterization of hemodialysis individuals who died after one year of this RRT, including their profile upon admission to this technique. We were able to identify factors associated with an increased risk of mortality within the first year. Finally, data on the majority of the variables were obtained from individual electronic clinical records that were regularly updated and easy to access.

## 5. Conclusions

To conclude, in this cohort of patients who started hemodialysis, a CHA2DS2-VASc score ≥ 3.5 and a central venous catheter at hemodialysis initiation predicted one-year mortality. We emphasize the need for additional studies in this population to assess this score as a predictor of mortality and to identify risk factors associated with an increased risk of mortality. Furthermore, it is imperative to develop strategies for the early identification and treatment of these high-risk patients, with the aim of delaying and/or reversing this unfavorable outcome.

## Figures and Tables

**Table 1:** Baseline characteristics.

Characteristic	Total (n = 856)	One Year Mortality (n = 152)	Survival (n = 704)	*p* Value
Age (year)	68.3 ± 15.5	76.8 ± 10.9	66.56 ± 15.7	<0.001
Gender (Male)—n (%)	524 (61.1)	93 (61.1)	429 (60.9)	0.955
Race (Caucasian)—n (%)	725 (84.5)	139 (91.4)	586 (83.2)	0.011
Comorbidities—n (%)				
Hypertension	770 (89.7)	130 (85.5)	638 (90.6)	0.061
Diabetes	392 (45.7)	68 (44.7)	324 (46.0)	0.773
Ischemic cardiomyopathy	197 (23.0)	52 (34.2)	145 (20.6)	<0.001
Atrial Fibrillation	137 (16.0)	31 (20.4)	106 (15.1)	0.104
COPD	89 (10.4)	20 (13.1)	69 (9.8)	0.219
Cerebrovascular disease	137 (16.0)	31 (20.4)	106 (15.1)	0.104
Peripheral artery disease	143 (16.7)	34 (22.3)	109 (15.4)	0.039
Dementia	43 (5.0)	13 (8.6)	30 (4.3)	0.028
Neoplasia	174 (20.3)	40 (26.3)	134 (19.2)	0.043
Chronic liver disease	35 (4.1)	9 (5.9)	26 (3.7)	0.210
Rheumatologic disease	72 (8.4)	15 (9.9)	57 (8.1)	0.475
CHA2DS2-VASc	3.7 ± 1.8	4.4 ± 1.7	3.5 ± 1.8	<0.001
Laboratory at dialysis start				
Haemoglobin (g/dL)	9.7 ± 1.6	9.7 ± 1.7	9.7 ± 1.6	0.706
Serum Urea (mg/dL)	196.7 ± 80.8	183.5 ± 77.2	199 ± 81.3	0.025
N/L ratio	5.6 ± 1.8	6.6 ± 1.9	5.4 ± 1.5	0.019
eGFR (ml/min/1.73 m^2^)	9.3 ± 5.8	10.6 ± 8.2	9.1 ± 5.1	0.004
Albumin (g/dL)	3.4 ± 0.7	3.1 ± 0.7	3.5 ± 0.7	<0.001
PTH (pg/mL)	352.9 ± 289.1	308.6 ± 295.4	361.3 ± 287.7	0.205
Ferritin (ng/mL)	527.8 ± 196.9	527.4 ± 282.5	527.9 ± 136.1	0.998
Vascular access at dialysis start—n (%)				
Central venous catheter	522 (60.9)	125 (82.2)	397 (56.3)	<0.001
Arteriovenous fistula	310 (36.2)	25 (16.4)	285 (40.4)	<0.001
Arteriovenous graft	24 (2.8)	2 (1.3)	22 (3.1)	0.220

**Table 2:** Patient characteristics according to their CHA2DS2-VASc score.

Characteristic	Total (n = 856)	CHA2DS2-VASc < 3.5 (n = 400)	CHA2DS2-VASc ≥ 3.5 (n = 456)	*p* Value
Age (year)	68.3 ± 15.5	59.9 ± 16.1	75.7 ± 10.4	<0.001
Gender (Male)—n (%)	524 (61.1)	287 (71.8)	237 (51.9)	<0.001
Race (Caucasian)—n (%)	725 (84.5)	306 (76.5)	419 (91.9)	<0.001
Comorbidities—n (%)				
Hypertension	770 (89.7)	333 (83.3)	437 (95.8)	<0.001
Diabetes	392 (45.7)	94 (23.5)	298 (65.4)	<0.001
Ischemic cardiomyopathy	197 (23.0)	43 (10.8)	154 (33.8)	<0.001
Atrial Fibrillation	137 (16.0)	6 (1.5)	131 (28.6)	<0.001
COPD	89 (10.4)	25 (6.3)	64 (14.0)	<0.001
Cerebrovascular disease	137 (16.0)	6 (1.5)	131 (28.6)	<0.001
Peripheral artery disease	143 (16.7)	17 (4.3)	126 (27.6)	<0.001
Dementia	43 (5.0)	3 (0.7)	40 (8.8)	<0.001
Neoplasia	174 (20.3)	84 (21)	90 (19.7)	0.624
Chronic liver disease	35 (4.1)	18 (4.5)	17 (3.7)	0.555
Rheumatologic disease	72 (8.4)	33 (8.3)	39 (8.6)	0.889
CHA2DS2-VASc	3.7 ± 1.8	2.1 ± 0.9	5.0 ± 1.1	<0.001
Laboratory at dialysis start				
Haemoglobin (g/dL)	9.7 ± 1.6	9.7 ± 1.6	9.7 ± 1.6	0.905
N/L ratio	5.6 ± 1.8	4.9 ± 2.7	6.2 ± 2.5	0.001
eGFR (ml/min/1.73 m^2^)	9.3 ± 5.8	8.7 ± 6.2	9.9 ± 5.4	0.006
Albumin (g/dL)	3.4 ± 0.7	3.5 ± 0.7	3.3 ± 0.7	<0.001
Central venous catheter at dialysis start—n (%)	522 (60.9)	213 (53.2)	309 (67.8)	<0.001
Outcomes				
One-year mortality—n (%)	152 (17.8)	43 (10.8)	109 (23.9)	<0.001

**Table 3:** Factors predictive of early mortality.

Characteristic	Mortality	*p*-Value	Adjusted HR (95% CI)
	Unadjusted HR (95% CI)		
Age	1.06 (1.04–1.08)	<0.001	
Gender (Male)	1.01 (0.71–1.45)	0.955	
Race (Caucasian)	2.15 (1.18–3.93)	0.013	1.43 (0.76–2.70)
Comorbidities			
Hypertension	0.61 (0.36–1.02)	0.611	
Diabetes	0.95 (0.67–1.35)	0.773	
Ischemic cardiomyopathy	2.00 (1.37–2.94)	<0.001	
Atrial fibrillation	1.42 (0.91–2.01)	0.101	
COPD	1.40 (0.82–2.37)	0.221	
Cerebrovascular disease	1.45 (0.93–2.26)	0.105	
Peripheral artery disease	1.57 (1.02–2.42)	0.040	
Dementia	2.10 (1.07–4.12)	0.032	1.18 (0.58–2.41)
Neoplasia	1.52 (1.01–2.28)	0.044	1.43 (0.93–2.22)
Chronic liver disease	1.64 (0.75–3.57)	0.214	
CHA2DS2-VASc	1.32 (1.20–1.46)	<0.001	
CHA2DS2-VASc >3.5	2.58 (1.76–3.78)	<0.001	2.24 (1.48–3.37)
Laboratory at dialysis start			
Haemoglobin	0.98 (0.88–1.09)	0.705	
Serum Urea	0.99 (0.99–1.00)	0.023	
N/L ratio	1.00 (1.00–1.06)	0.027	1.00 (1.00–1.00)
eGFR	1.04 (1.01–1.07)	0.007	1.01 (0.98–1.04)
Albumin	0.40 (0.30–0.52)	<0.001	1..03 (1.00–1.06)
PTH	1.00 (1.00–1.00)	0.208	
Ferritin	1.00 (1.00–1.00)	0.998	
Central venous catheter at dialysis starts	3.58 (2.30–5.57)	<0.001	3.06 (1.93–4.85)

## Data Availability

Not applicable.
